# Comparative Variation within the Genome of *Campylobacter jejuni* NCTC 11168 in Human and Murine Hosts

**DOI:** 10.1371/journal.pone.0088229

**Published:** 2014-02-07

**Authors:** Dallas K. Thomas, Abdul G. Lone, L. Brent Selinger, Eduardo N. Taboada, Richard R. E. Uwiera, D. Wade Abbott, G. Douglas Inglis

**Affiliations:** 1 Agriculture and Agri-Food Canada Research Centre, Lethbridge, Alberta, Canada; 2 Department of Biological Sciences, University of Lethbridge, Lethbridge, Alberta, Canada; 3 Public Health Agency of Canada, Lethbridge, Alberta, Canada; 4 Department of Agricultural, Food and Nutritional Science, University of Alberta, Edmonton, Alberta, Canada; University of Illinois at Chicago College of Medicine, United States of America

## Abstract

Campylobacteriosis incited by *C. jejuni* is a significant enteric disease of human beings. A person working with two reference strains of *C. jejuni* National Collection of Type Cultures (NCTC) 11168 developed symptoms of severe enteritis including bloody diarrhea. The worker was determined to be infected by *C. jejuni*. In excess of 50 isolates were recovered from the worker’s stool. All of the recovered isolates and the two reference strains were indistinguishable from each other based on comparative genomic fingerprint subtyping. Whole genome sequence analysis indicated that the worker was infected with a *C. jejuni* NCTC 11168 obtained from the American Type Culture Collection; this strain (NCTC 11168-GSv) is the genome sequence reference. After passage through the human host, major genetic changes including indel mutations within twelve contingency loci conferring phase variations were detected in the genome of *C. jejuni*. Specific and robust single nucleotide polymorphism (SNP) changes in the human host were also observed in two loci (Cj0144c, Cj1564). In mice inoculated with an isolate of *C. jejuni* NCTC 11168-GSv from the infected person, the isolate underwent further genetic variation. At nine loci, mutations specific to inoculated mice including five SNP changes were observed. The two predominant SNPs observed in the human host reverted in mice. Genetic variations occurring in the genome of *C. jejuni* in mice corresponded to increased densities of *C. jejuni* cells associated with cecal mucosa. In conclusion, *C. jejuni* NCTC 11168-GSv was found to be highly virulent in a human being inciting severe enteritis. Host-specific mutations in the person with enteritis occurred/were selected for in the genome of *C. jejuni*, and many were not maintained in mice. Information obtained in the current study provides new information on host-specific genetic adaptation by *C. jejuni*.

## Introduction

Campylobacteriosis incited by *C. jejuni* is a prevalent enteric disease of people. For example, we recently observed that 10.3% of 2251 diarrheic individuals were culture positive for *C. jejuni* over a 1 year period in southwestern Alberta, Canada (unpublished). The epidemiology of campylobacteriosis is poorly understood at present, and the source attribution hypothesis suggests that some genetic lineages of *C. jejuni* exist that define host adaptation. This hypothesis is consistent with observations that certain genetic lineages of *C. jejuni* are commonly associated with diarrheic human beings but not with non-human hosts [1]. The application of multilocus sequence typing in conjunction with source attribution models has been used to link various reservoirs of *C. jejuni* with human infections [2]–[6]. However, the degree to which host specificity exists across various sub-lineages (i.e. Sequence Types or STs) in the *C. jejuni* population is unclear. For example, Gripp et al. [7] observed considerable phenotypic diversity within ST-21 which did not correspond to isolation source; the genetic diversity observed was attributed to recombination and gain of phage-related genes. Moreover, although there is considerable source specificity for many STs, there are many highly prevalent STs for which multiple sources are possible. Whether genotyping schemes with higher resolution such as comparative genomic fingerprint (CGF) subtyping [1], [8] will yield data with improved host specificity remains to be determined.

*Campylobacter jejuni* National Collection of Type Cultures (NCTC) 11168 was originally isolated from the feces of a diarrheic human being in 1977 [9], and it is the genomic reference strain (NCTC 11168-GS) for the species [10]. Similarly to other *C. jejuni* strains, NCTC 11168 contains many homopolymeric tracts of nucleotides within its genome [10], [11]. These regions are hypervariable and function as contingency loci that frequently undergo slipped-strand mispairing during replication resulting in insertions and/or deletions (i.e. indels) and subsequent frame shifts and phase variation [12]. This phenomenon is thought to be important in host adaptation, enhanced virulence, and immune evasion via rapid changes in cell surface characteristics [10], [13], [14]. It has long been recognized that repeated culturing or passage through animal hosts results in phenotypic changes in microorganisms including *C. jejuni* [15]–[18]. In this regard, several genetic variants of *C. jejuni* NCTC 11168 have been characterized [19], [20] including variants that possess different phenotypic characteristics relative to the parent strain [21], [22]. Recent reports have shown that the genome of *C. jejuni* undergoes rapid microevolutonary change during passage through the gastrointestinal tracts of poultry and/or murine hosts [23]–[25]. It is now recognized that phase variation to modify gene expression as an alternative to conventional genetic regulatory mechanisms may play an important role in the success of *C. jejuni* as an enteric pathogen [23]–[27]. Whether similar genetic change occurs within immunocompetent hosts in which the bacterium incites disease such as human beings is currently uncertain. It is noteworthy that although NCTC 11168 was originally isolated from a diarrheic human being, its virulence in people has not been examined. We hypothesize that *C. jejuni* NCTC 11168 will undergo rapid and host-specific genetic changes in an immunocompetent human host with enteritis. To test this hypothesis, we comparatively examined the degree of mutations within the genome of *C. jejuni* NCTC 11168 in an infected person in which the bacterium incited severe acute inflammation, and the stability of genetic change in an alternate mammalian host (i.e. mice).

## Materials and Methods

### Ethics Statement

The stool sample of the person infected by *C. jejuni* was donated by the afflicted individual. Written informed consent was provided by the infected individual to isolate *C. jejuni* from their stool sample and to genotype and utilize the recovered *C. jejuni* isolates in subsequent research. The component of the study involving the use of interleukin (IL)-10 knockout (KO) mice was carried out in strict accordance with the recommendations specified in the Canadian Council on Animal Care Guidelines. The project was reviewed and approved by the Lethbridge Research Centre (LRC) Animal Care Committee (Animal Use Protocol Review 0703) and the LRC Biosafety and Biosecurity Committee before research commenced.

### Isolates

*Campylobacter jejuni* NCTC 11168-GSv was obtained from American Type Culture Collection (ATCC 700819). This strain is the genome reference strain for the species [10]. *Campylobacter jejuni* NCTC 11168-V26 was obtained from Dr. Brenda Allen (Vaccine and Infectious Disease Institute, Saskatoon, SK). This strain was originally obtained from ATCC by the National Research Council Canada in 1977, and it was repeatedly laboratory-passaged; the strain was subsequently determined to differ both phenotypically and genetically from NCTC 11168-GS and was designated as a variant strain [22].

In late October 2003, a person working with *C. jejuni* isolates developed severe diarrhea. On October 30, the individual sought medical assistance, and a stool sample was submitted to the Microbiology Diagnostic Laboratory (MDL) at Chinook Regional Hospital located in Lethbridge, Alberta, Canada. The unused stool sample was stored at 4°C. The afflicted individual was subsequently diagnosed with campylobacteriosis incited by *C. jejuni* in early November by MDL staff. No other enteric pathogens were detected. The stool sample submitted to the MDL in Cary-Blair transport medium [28] was obtained. A subsample of the stool was suspended in sterile Columbia broth (Oxoid, Thermo Scientific, Nepean, ON), streaked onto Karmali agar (Oxoid) containing selective supplement SR167 (Oxoid), and cultures were maintained at 40°C in a microaerobic environment (10% CO_2_, 3% H_2_, 5% O_2_, 82% N_2_). After 48 hr, multiple colonies (>50 isolates) were recovered (maximum of two isolates per culture), streaked for purity on Karmali agar, and biomass for each isolate was stored in Columbia broth containing 30% glycerol at −80°C (with no subculturing).

### Isolate Characterization

All isolates were tested for their ability to hydrolyze hippurate [29], and DNA was extracted and subjected to taxon-specific PCR for *Campylobacter* genus (16 S rRNA gene) and *C. jejuni* (*hip*O and *map*A genes) [30]–[32]. The near complete 16 S rRNA gene of arbitrarily-selected isolates was also sequenced, and the sequences were compared to reference sequences in the NBCI using BlastN. All recovered isolates from the human stool, as well as NCTC 11168-GSv and NCTC 11168-V26 were genotyped using the CGF40 subtyping method [1].

### Murine Intestinal Colonization

Mice (C57BL/6J IL-10^−/−^) were obtained from a breeding colony maintained at LRC. Mice were originally purchased from The Jackson Laboratory (Bar Harbor, ME). All mice were maintained in a germ-free environment, were fed autoclaved AIN-93G Purified Rodent feed (Sterigenics, Rockaway NJ) and water *ad libitum*, and were monitored for behavioural and physiological signs of distress. Before inoculation, feces from all mice were aseptically collected and examined for the presence of *C. jejuni*. Feces were homogenized in Columbia broth, a 100 µl aliquot of the suspension was spread on Karmali agar containing selective supplement SR167, and cultures were maintained at 40°C in a microaerobic environment for 72 hr and examined for the presence of *C. jejuni* colonies. All mice were deemed free of *C. jejuni*.

A single arbitrarily-selected *C. jejuni* isolate from the stool of the infected person (K12E5) was grown on Columbia agar amended with 5% sheep blood and selective supplement SR167 (Columbia blood agar) at 40°C for 16 hr. Biomass was carefully removed from the surface of the agar medium, and cells were suspended in phosphate buffered saline (PBS; pH 7.2). The density of cells was adjusted to 2×10^9^ cells ml^−1^. Three mice were gavage inoculated with 100 µl of the cell suspension within 30 min of the collection of *C. jejuni* cells. To confirm densities of viable cells, inoculum was diluted in a 10-fold dilution series, 100 µl of each dilution was spread in duplicate onto Karmali agar, cultures were incubated at 40°C in a microaerobic atmosphere, and the number of *C. jejuni* colonies were counted at the dilution yielding 30 to 300 colony forming units (CFU) after 48 hr of incubation. Aliquots of the inoculum were also examined microscopically for the presence of highly motile *C. jejuni* cells (i.e. before and after the 30 min inoculation period). After inoculation of mice, feces were collected commencing 2 and 7 days post-inoculation (p.i), and at 7 day intervals thereafter up to 28 days p.i. Densities of *C. jejuni* cells within feces were determined by dilution spread-plating on Karmali agar containing selective supplement SR167 as above, and CFU g^−1^ of feces were determined. On day 28, the mice were anesthetized with isofluorane (Halocarbon Products Corporation, River Edge, NJ), and humanely euthanized under anaesthesia by cervical dislocation. The intestinal tract of each mouse was exposed, and the cecum was aseptically excised. The cecum of each mouse was opened longitudinally, mucosal surfaces were gently washed with chilled sterile PBS, and biopsies (5–10 mg) were removed for DNA extraction. The mucosal surfaces of five cecal pieces were rubbed over the surface of Columbia blood agar, and cultures were maintained at 40°C in a microaerobic environment. After 16 hr, growth of *C. jejuni* on the medium was confirmed by colony morphology, as well as cell morphology and motility. Cells were harvested from the medium surface, and suspended in PBS. The density of cells was adjusted 2×10^9^ cells ml^−1^, and equal volumes of the adjusted cell suspension from each mouse were combined. Three *C. jejuni*-free mice were gavage inoculated with the fresh cell suspension, mice were maintained, and samples were collected and processed as above. The procedure was repeated for a total of three passages. From last group of mice, *C. jejuni* colonies from ceca and feces at 28 days p.i. were collected from the surface of Columbia blood agar, and biomass was stored at −80°C; care was exercised to ensure the purity of the *C. jejuni* biomass collected.

### Quantitative PCR

Densities of *C. jejuni* associated with mucosa of the jejunum, ileum, cecum, and descending colon were determined by quantitative PCR targeting the *map*A gene [33]. Genomic DNA was extracted from tissue using a RTP Bacteria DNA Mini Kit (Invitek, Berlin, Germany) according to the manufacturer’s instructions. Concentrations of DNA were quantified using a fluorometer (TD360; Turner Designs, Sunnyvale, CA), and DNA was stored at −80°C. The SYBR Green-based standard curve method for quantification of DNA was carried out using Power SYBR® Green PCR (Life Technologies, Carlsbad, CA). Each 20 µl PCR reaction contained 2 µl of DNA (20–50 ng), 10 µl of the 2X Power SYBR® Green PCR Master Mix, and 200 nmol of each of the forward and reverse primers. The QCjmapANF and QCjmapANR primers were used. A standard curve was established using genomic DNA from *C. jejuni* NCTC 11168-GSv; DNA copy number varied from 10^1^ to 10^7^. Samples were amplified as follows: one cycle at 95°C for 10 min; and 40 cycles at 95°C for 15 sec, and 60°C for 60 sec. A Stratagene Mx 3005 p (Stratagene Products, La Jolla, CA) was used. Each PCR reaction per sample was run in triplicate, and the mean value was calculated. The number of *C. jejuni* cells was expressed as copy number mg^−1^ of tissue. For all reactions, melt curve analysis was conducted to confirm amplification specificity.

Data were analyzed using the MIXED procedure of SAS (SAS Institute Inc., Cary NC). Intestinal location was treated as a repeated measure, and the appropriate covariance structure was utilized according to the lowest Akaike’s Information Criterion (AIC). In conjunction with a significant F test, the lsmeans function of SAS was used to compare location and passage.

### Illumina Sequencing

The *C. jejuni* samples sequenced were: (A) NCTC 11168-GSv; (B) NCTC 11168-GSv after four culturing cycles; (C) Kf1 consisting of the collective genomic DNA of 52 individual isolates recovered from the stool of the infected person (replicate 1); (D) Kf2 consisting of the collective genomic DNA of 52 individual isolates recovered from the stool of the infected person (replicate 2); (E) a single isolate, K12E5 recovered from the infected person; (F) mcK12E5 consisting of genomic DNA of *C. jejuni* isolates recovered from ceca after three passages through mice; (G) mfK12E5 consisting of the collective genomic DNA of *C. jejuni* isolates recovered from murine feces after three passages through mice; and (H) NCTC 11168-V26. Upon arrival of reference isolates at LRC (i.e. sample A and H) or after initial isolation, all isolates were maintained at −80°C with no sub-culturing. To obtain biomass for genomic DNA extraction, isolates were cultured on Columbia blood agar, cultures were maintained at 40°C in a microaerobic environment for 24 hr, cells were carefully removed from the surface of the medium and suspended in PBS, cells were pelleted by centrifugation (16,000×g), and the supernatant removed. Genomic DNA was extracted using the RTP Bacteria DNA Mini Kit (Invitek) according to the manufacturer’s instructions. Concentrations of DNA were quantified using a fluorometer (Turner Designs). For samples C and D, equal quantities of DNA from each of the 52 isolates were combined into a master mix, and the composite sample was divided into two equal aliquots for analysis. The final concentration of genomic DNA in samples ranged from 20–50 ng µl^−1^. Barcoded libraries for Illumina sequence were constructed at the Michael Smith Genome Sciences Centre (University of British Columbia, Vancouver, BC). Paired-end sequence data (75 bp read lengths) was obtained using an Illumina HiSeq 2000 (Illumina Inc., San Diego, CA) located at the Genome Sciences Centre.

### Bioinformatic Analyses

Illumina data for each sample was received as paired-end FastQ data files. Prior to analysis, data for individual samples was processed through an in-house quality control (QC) pipeline which resulted in an approximate 15–20% decrease in total raw sequences. The QC pipeline integrated a cleaning phase comprised of artifact filtering, adapter removal, and low complexity screening. Quality control data for each sample were aligned to the *C. jejuni* NCTC 11168-GS reference genome obtained from GenBank, National Center for Biotechnology Information using the Bowtie2 aligner [34]. Aligned data was further processed into a position-based alignment output using SAMTools [35]. Further analysis was conducted in three separate phases. In phase one, identification of the reference strain infecting the human worker was ascertained. In phase two, comparative genomic analysis of strains was conducted against NCTC 11168-GS. In phase three, a 70 times coverage subsample was extracted from each data sample and a mapping assembly was performed using the MIRA assembler [36]; assembled results were used to validate results of phase two. Circos [37] was used to visualize genomic data in a circular layout to facilitate strain identification. Modified alignment data for samples K12E5, NCTC 11168-GSv, and NCTC 11168-V26 were mapped relative to the annotated genome of NCTC 11168-GS. In-house scripts were used to parse the alignment data for use by Circos. Potential single nucleotide polymorphisms (SNPs) and indels from the alignment output were extracted and parsed using scripts developed in-house. A quality score of 15 (an error rate of approximately 1 in 50) and a frequency delineator of 15% (i.e. delimiting true genetic change from sequence variation) were applied. Genetic variations at specific loci were first validated against the mapping assembly results and then compared with the annotated data for NCTC 11168-GS, and amino acid substitutions due to SNPs and potential frame shifts created by indels were identified.

The predicted structure of the C-terminus (residues: 135–260) of Cj1087c was generated using Phyre2 [38]. The intact and truncated sequences were threaded to a model of LytM (Pdb ID: 2B0P, [39]) with an E-value of 3.2 e^−22^ with 100% estimated precision, and 5.5 e^−21^ with 100% estimated precision, respectively. The models were aligned with WinCoot [40] and visualized with PyMOL (Available www.pymol.org. Accessed 2013 Aug 02).

### Accession Numbers

Whole genome sequences associated with this manuscript were deposited in GenBank under the accession numbers CP006685, CP006686, CP006687, CP006688, and CP006689 for K12E5, Kf1, mcK12E5, mfK12E5, and NCTC 11168-GSv, respectively.

## Results and Discussion

### Infection of a Human Being

On October 25, 2003 an individual working with cultures of *C. jejuni* on October 23 developed a high fever, vomiting, severe headache, myalgia including a sore neck, bloody diarrhea, dehydration, and blood in urine. Meningitis was presumptively diagnosed and Novo-Doxylin (doxycycline) and apo-amitriptyline were initially prescribed. Blood and cerebrospinal fluid (obtained from a lumbar puncture) were culture negative, but a stool sample submitted for diagnosis was culture positive for *C. jejuni*. No other enteric pathogens were detected from the stool. Erythromycin was prescribed and the person fully recovered from the infection. The stool sample submitted for diagnosis was maintained at 4°C. On November 4, the stool sample was transferred to Agriculture and Agri-Food Canada (10 days after submission). In excess of 50 presumptive *Campylobacter* isolates were recovered. All isolates were hippurate positive and were identified as *C. jejuni* by taxon-specific PCR. CGF40 subtyping revealed that the isolates were indistinguishable from each other, and from NCTC 11168-GSv and NCTC 11168-V26; the isolates from the stool and reference strains were positive for all 40 loci within the CGF assay. With the exception of NCTC 11168-GSv and NCTC 11168-V26, all other strains of *C. jejuni* that the individual may have had been potentially exposed to up to 7 days prior to the onset of symptoms/signs of campylobacteriosis possessed conspicuously different CGF patterns (data not presented). Thus, the afflicted individual was deemed to have been infected with either NCTC 11168-GSv or NCTC 11168-V26.

### Whole Genome Strain Identification

**Figure 1:**
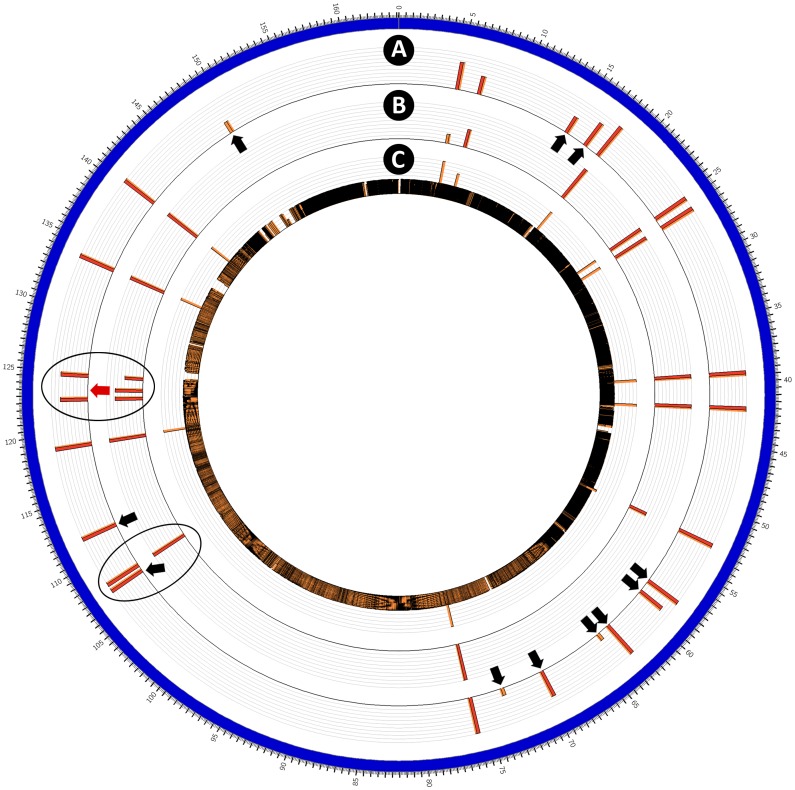
Circular representation of the genome of *Campylobacter jejuni* NCTC 11168. Genome maps (in order of presentation from outside to inside) are: (A) K12E5 from an infected human being; (B) NCTC 11168-GSv; and (C) NCTC 11168-V26. The scale on the outside of the outermost map represents genome location (x 10^4^ bases). Red and orange bars represent mutations relative to the original annotated reference NCTC 11168-GS strain deposited in GenBank (AL111168.1) [10], [13]. The height of the red and orange bars indicates mutation frequency. Ovals denote mutations common to NCTC 11168-GSv and K12E5, but not NCTC 11168-V26. Black arrows denote mutations present in K12E5 but not in NCTC 11168-GSv, and red arrows the converse. See Table 1 for additional information.

Comparative Illumina whole genome sequence analysis revealed a high degree of similarity between an arbitrarily-selected isolate from the infected individual (i.e. K12E5) and NCTC 11168-GSv. A large number of SNPs and indels unique to NCTC 11168-V26 were observed indicating that the afflicted individual was infected with NCTC 11168-GSv and not NCTC 11168-V26 (Figure 1). Although *C. jejuni* NCTC 11168 was originally isolated from the feces of a diarrheic patient [9], and it represents the sequence reference strain for *C. jejuni* [10], its virulence in human beings was uncertain. The severity of the symptoms observed in the individual infected with NCTC 11168-GSv in the current study, including vomiting, severe bloody diarrhea, dehydration, fever, myalgia, and blood in urine indicative of hemolytic uremic syndrome (HUS) indicate that NCTC 11168-GSv is highly virulent in human beings. Acute diarrhea, dehydration, fever, and myalgia are common symptoms of campylobacteriosis [41], [42]. Vomiting and colitis with ensuing bloody diarrhea occurs in a subset of human beings infected by *C. jejuni* [43]–[45]. In severely diarrheic human beings, *C. jejuni* infection is characterized by histopathologic evidence of inflammation (e.g. infiltration of inflammatory cells, abnormal mucus gland morphology, crypt abscesses, mucosal ulceration), and the presence of large numbers of polymorphonuclear leukocytes in stools [46]. A creatinine test indicated that the kidneys of the infected individual had been affected. Although rare, HUS has been observed in some individuals infected by *C. jejuni* in the absence of other enteric pathogens [47]–[49]. Recently, glomerulonephritis was also observed in a human infected with *C. jejuni* [50], but the occurrence/extent of nephritis in the human being infected with *C. jejuni* NCTC 11168-GSv in the current study is unknown. Genetic factors contributing to human pathogenicity/virulence in *C. jejuni* are very poorly understood at present. Hemolytic uremic syndrome has been linked to the secretion of a toxin by other enteric bacteria such as enterohemorrhagic *Escherichia coli* [51], but no gene homologs for a Shiga-like toxin are present in the genome of NCTC 11168-GS or any other strain of *C. jejuni*. To date, the cytolethal distending toxin (CDT) encoded by the CDT operon is the only verified toxin produced by *C. jejuni* [52]; although its role in pathogenesis has not been fully resolved, there is no evidence that the CDT is involved in HUS. Despite the genetic and phenotypic changes that have occurred in *C. jejuni* NCTC 11168-GS relative to NCTC 11168-OS (i.e. original strain deposited in NCTC), the severe symptoms incited by NCTC 11168-GSv in the afflicted person in the current study, including the impact of the bacterium on the person’s kidneys confirms that this strain is highly virulent and an appropriate *C. jejuni* genotype for pathogenicity studies. Although *C. jejuni* NCTC 11168-GSv is highly virulent in human beings, the strain is not as effective a colonizer of the gastro-intestinal tract of chickens as other strains [53], and this raises questions as to its colonization potential in other hosts including non-avian reservoirs of human infectious *C. jejuni* such as ruminants.

### Genetic Variation in a Human Host

To our knowledge, the current study is the first to examine genetic changes in *C. jejuni* that occur during human infection. Of note, the afflicted human subject was a fully immunocompetent individual, who became infected with NCTC 11168-GSv, which provided the opportunity to directly compare our results to those observed for this strain in other hosts. Host-specific changes to the genome of *C. jejuni* have recently been observed in a number of non-human hosts including mice and chickens [23]–[25]. A similar Illumina sequencing strategy to that described by Jerome et al. [25] in mice was employed in the current study. To ascertain the degree to which genetic variation occurred in a human host with enteritis, the collective genome sequence of 52 isolates of *C. jejuni* recovered from the stool of a person infected with NCTC 11168-GSv (i.e. Kf1 and Kf2) was compared to the whole genome sequence of NCTC 11168-GS. To address reproducibility, two independent sequencing runs (separate aliquots of DNA from same collective group of isolates) were conducted for *C. jejuni* isolates recovered from the infected human. The two runs provided very similar results across the entire genome (≤5.6% difference in mutation frequency amongst variable genes) indicating that the Illumina sequencing results were highly reproducible. The application of high-coverage next-generation sequencing allowed us to identify frequency changes with a high degree of resolution within a population of *C. jejuni* cells across the entire genome.

Major variations between NCTC 11168-GSv and Kf1/Kf2 were observed in both sequencing runs at 15 loci (i.e. Cj0031, Cj0144, Cj0170, Cj0262c, 527378, Cj0617, Cj0628, Cj0676, Cj0685c, 695943, Cj1139c, Cj1190c, Cj1295, Cj1305c, Cj1564) (Table 1), eleven of which were indels. All 11 indels occurred in genes possessing homopolymeric regions. These genes encode the following putative proteins: a type IIS restriction/modification enzyme (Cj0031); a potassium transporter protein (Cj0676); a sugar transferase invasion protein (Cj0685c); a β-1,3 galactosyltransferase (Cj1139c); and four unknown hypothetical proteins (Cj0170, Cj0617, Cj0628, Cj1295, Cj1305c). The remaining indels occurred in intergenic regions (527378, and 695943).

**Table 1:** Genomic changes in *C. jejuni* NCTC 11168-GSv *in vitro* and *in vivo* (relative to the sequence data for NCTC 11168-GS in GenBank [10]).

#	Locus	Alteration^a^	Type	Sample (variant/frequency)^b^						
				A^c^	B	C	D	E	F	G
1	Cj0031^d^	R858R	−1G	−	−	+	+	+	+	+
				7.1	9.4	30.4^i^	30.6^i^	31.6	34.3	36.5
2	Cj0046^d^	–f	−1G	+	+	+	+	+	−	−
				19.0	28.1	25.3	26.7	25.0	0.0	0.0
	Cj0046c	–f	−2G	−	−	−	−	−	+	+
				0.0	0.0	0.0	0.0	0.0	5.8^k^	6.7^k^
3	Cj0144c	E508K	G/A	−	−	+	+	+	−	−
				0.0	0.0	52.9^i^	51.3^i^	52.6	1.1^k^	0.0^k^
4	Cj0170^d^	G082G	−1G	−	−	+	+	+	−	−
				0.0	1.3	27.0^i^	29.6^i^	28.3	1.7^k^	4.0^k^
5	Cj0184c	V384I	−2AC	+	+	+	+	+	+	+
				50.6	46.1	45.4	41.9	42.4	42.5	44.9
6	Cj0262c	E514K	C/T	−	−	+	+	−	−	−
				0.0	0.0	0.0	0.0	0.0	0.0	0.0
7	Cj0276	D048G	A/G	+	+	+	+	+	+	+
				100.0	100.0	100.0	99.8	100.0	100.0	100.0
8	Cj0284c	I290T	A/G	+	+	+	+	+	+	+
				100.0	99.8	100.0	99.9	100.0	99.9	99.8
9	Cj0431	g205K	T/A	+	+	+	+	+	+	+
				100.0	100.0	100.0	99.9	100.0	100.0	100.0
10	Cj0455c	g115Q	A/G	+	+	+	+	+	+	+
				100.0	100.0	100.0	99.9	99.9	100.0	100.0
11	Cj0548	T629I	C/T	−	−	−	−	−	+	−
				0.0	0.0	0.0	0.0	0.0	99.8	0.0
12	527378^de^	–f	−1G	+	+	−	−	−	−	−
				11.4	8.4	0.0	0.0	0.0	0.0	0.0
12	527378^de^	–f	−2G	−	−	−	−	−	+	+
				0.0	0.0	0.0	0.0	0.0	15.7^k^	17.1^k^
12	527378^de^	–f	−3G	−	−	+	+	+	−	−
				0.0	0.0	29.6^i^	29.9^i^	33.6	0.0	0.0
13	Cj0617^d^	V190G or	+1G	−	−	+	+	+	+	+
		R187R		0.0	2.6	23.2^i^	22.9^i^	27.8	30.0	26.1
14	Cj0628^d^	G168G	+1G	−	−	+	+	+	+	+
				0.0	0.4	13.8	13.0	16.3	13.8	8.9
15	Cj0676^d^	K284K	+1G	−	−	+	+	+	+	+
				0.0	0.6	27.9^i^	25.6^i^	30.5	32.4	33.2
16	Cj0685c^d^	H295I	−1G	-	-	+	+	-	-	-
				0.0	0.0	27.9^i^	29.5^i^	4.1^j^	0.5	1.1
17	695943^de^	–f	+1C	−	−	+	+	+	+	+
				27.6	30.6	26.5	27.8	28.5	28.6	26.5
18	Cj0807	K198E	A/G	+	+	+	+	+	+	+
				100.0	99.7	100.0	99.9	100.0	100.0	100.0
19	Cj1087c	G232G/I231G	−12 CGTCAACTATCC	−	−	−	−	−	+	+
		L230G/Q229G		0.0	0.0	0.0	0.0	0.0	30.2	30.1
20	Cj1139c^d^	G113G	+1C	−	−	+	+	+	+	+
				0.0	0.4	38.2^i^	40.2^i^	36.8	40.8	37.9
21	Cj1145c^d^	V002G	+1C	+	+	+	+	+	+	+
				33.3	26.8	25.9	27.3	33.7	28.8	36.3
22	Cj1190c	V204A	A/G	−	−	−	−	−	+	+
				0.0	0.0	18.2	18.4	0.2	99.8	100.0
22	Cj1190c	R101T	C/G	−	−	+	+	−	+	+
				0.0	0.2	99.8^i^	99.9^i^	91.7	99.8	99.8
23	Cj1259	E180G	A/G	+	+	+	+	+	+	+
				100.0	100.0	100.0	100.0	100.0	99.9	100.0
24	Cj1295^d^	I051Y or	+1G	+	+	+	+	+	+	+
		L048L		34.6	19.3^h^	16.0^i^	19.8	22.4	15.4	6.4
25	Cj1305c^d^	S197V	+1C	+	+	−	−	−	+	−
				42.6	29.3	3.9^i^	2.8^i^	0.5	22.2^k^	16.6^k^
26	Cj1318^d^	G059V	−1G	+	+	+	+	+	+	+
				23.5	17.9	18.6	19.3	17.5	22.4	20.0
27	1338389^e^	–f	+1A	+	+	+	+	+	+	+
				35.3	42.1	41.6	41.6	42.9	41.4	37.9
28	Cj1470c	M269U	+1T	+	+	+	+	+	+	+
				44.4	34.7	48.4	42.8	42.4	40.4	39.1
29	Cj1564	E511K	A/G	−	−	+	+	+	−	−
				0.0	0.3	49.0^i^	47.3^i^	47.9	0.3^k^	0.7^k^

a Alteration location where the first letter indicates the original amino acid, the second letter indicates the substituted amino acid, and the number indicates the position at which the amino acid substitution occurred.

b Frequency data denotes potential variants as determined through Multiple-Sequence alignment. +/− data denotes validated variants determined through a Mapping-Assembly using MIRA.

c *Campylobacter jejuni* genomes: (A) NCTC 11168-GSv; (B) NCTC 11168-GSv after four transfers *in vitro*; (C) Kf1 [collective isolates-human host passage]; (D) Kf2 [collective isolates-human host passage]; (E) K12E5 [single isolate-human host passage]; (F) mcK12E5 [collective isolates from the ceca of mice after three passages]; and (G) mfK12E5 [collective isolates from feces of mice after three passages].

d Denotes a hypervariable region within the genome of NCTC 11168-GS.

e Locus unknown.

f No protein information available.

g Stop codon.

h ≥15% difference in A *versus* B.

i ≥15% difference in C or D *versus* A (i.e. genetic variation due to human passage).

j ≥15% difference in E *versus* the mean of C and D (i.e. genetic variation due to human passage).

k ≥15% difference in F or G versus E consistent with C and D (i.e. genetic variation due to murine passage).

The occurrence of indel mutations within homopolymeric regions of the genome of *C. jejuni* NCTC 11168-GSv in human beings might be expected based on previous observations in IL-10 KO mice [25]. Mutability in contingency loci is now believed to be a very important strategy utilized by *C. jejuni in vivo* (i.e. adaptive microevolution). As such, we also observed changes in phase frequencies that appeared to be modulated by the host via selection, and the retention of a background population of variants due to the stochasticity of slipped-strand mutation [25]. Seven of the indel mutations that we detected were within homopolymeric regions of the *C. jejuni* NCTC 11168-GSv genome in the human being (i.e. Cj0031, Cj0170, Cj0617, Cj0685c, Cj1139c, Cj1295, Cj1305c) have been previously reported in chickens and/or mice [23], [25]. Furthermore, the indel occurring in Cj0676 has been previously observed *in vitro* [20]. The biological relevance of the high frequency occurrence of the indels within intergenic regions is uncertain. Of the loci affected, only Cj1295 has been characterized, and is associated with flagellin glycosylation [54]. The biological relevance of the indel mutations that we observed in contingency loci in the human being is uncertain. Jerome et al. [25] suggested that many indel mutations in contingency loci play an important role in host immune evasion, but it is highly likely that indels are associated with other aspects of host colonisation and/or pathogenesis as well. Our data also confirm the importance of considering *C. jejuni* as a dynamic population comprised of multiple genotypes and phenotypes that undergo rapid diversification through *in vivo* and *in vitro* selection, as opposed to a clonal entity.

In addition to indel mutations, we observed point mutations in three loci (i.e. Cj0144, Cj0262c, Cj1564) (Table 1). Of note, Cj0262c was observed in the mapping assembly but not in the alignment. Two of the SNPs observed in the genome of *C. jejuni* NCTC 11168-GSv (i.e. Cj0144, Cj1564) after passage through the human host were robust with a ≥47 fold increase in frequency. The SNPs occurred in genes that encode putative methyl-accepting chemotaxis signal transduction proteins. For each of Cj0144, Cj0262c, and Cj1564, the SNPs observed would confer an amino acid substitution. To our knowledge these SNPs have not been previously observed. It is possible that these SNPs are human host specific; comparative genomic research *in vivo* has to date been restricted to asymptomatic hosts such as chickens and immune compromised murine models of inflammation (IL-10 KO mice). Based on the lack of any SNPs, duplications, insertions, or other polymorphisms in *C. jejuni* NCTC 11168 in IL-10 KO mice, Jerome et al. [25] concluded that contingency genes are the primary driver of adaptive microevolution *in vivo*. For SNPs to be biologically-relevant *in vivo*, they should become fixed in the genome. As it was not possible for us to evaluate this possibility in human subjects, we passaged *C. jejuni* NCTC 11168 from the infected human through IL-10 KO mice. The SNPs observed in *C. jejuni* NCTC 11168 after passage through the human being were not fixed and reverted in mice. The relevance of the reversions is uncertain. One possibility is that these SNPs are host-specific (i.e. to human beings ± inflammation). *C. jejuni* has been documented to induce inflammation in IL-10 KO mice (i.e. as an inflammation model) [55]–[59]. However, we have never observed inflammation in IL-10 KO mice inoculated with a multitude of *C. jejuni* strains, and we did not observe any inflammation in IL-10 KO mice inoculated with NCTC 11168-GSv in the current study. Reasons for this discrepancy relative to other research groups are unknown. However, the host-pathogen-microbiota interaction is exceptionally complex, and it is now recognized that the intestinal microbiota and the conditions in which mice are raised and housed influence disease development [60]. Indeed, genetically modified mice that are either raised as germ free or specific pathogen free (SPF) animals (i.e. similar to the SPF IL-10 KO mice used in the current study) will exhibit a muted inflammatory response in the intestine [61], [62]. Thus, the SNPs that we observed are either specific to the human being infected in the current study and/or that Th1 inflammation is required for selection. IL-10 is a cytokine involved in both innate and adaptive immune responses and it is expressed by many cell types including macrophages, dendritic cells, granulocytes, and B and T lymphocytes [63]–[67]. Although the use of IL-10 deficient mice has proven instrumental in elucidating mechanisms of enteritis [68], IL-10 deficient animals cannot mount a balanced T helper-associated inflammatory cascade. Most certainly, the inflammatory response in IL-10 KO mice may not represent responses that occur in conventionally raised, wild-type mice or in non-rodent species in all instances. As such, 1L-10 KO mice may not mimic the normal immune or inflammatory responses mounted by people.

A limitation of our study was that our examination was restricted to a single strain of *C. jejuni*. Laboratory-acquired infections by *C. jejuni* unfortunately occur [69]. When they are encountered, researchers should not only identify the strain responsible for the infection, but also should record the characteristics of the infection (e.g. symptoms, signs and severity), and recover and store as many isolates as possible from the infected person. Subsequent comparative genomic analyses for a multitude of *C. jejuni* strains infecting human beings via laboratory-acquired infections may provide valuable insight on adaptation/pathogenesis. A similar approach should be applied for inadvertent non-laboratory-acquired infections of human beings by *C. jejuni* (e.g. from animal sources) for which the isolate source can be identified [70]. However, this approach is more difficult as definitively ascertaining the strain responsible for the infection can be very challenging; commonly-used genotyping methods for *C. jejuni* may lack the resolution and throughput to definitively identify the responsible strain.

### Genetic Variation *in vitro*

It is generally accepted that repeated culturing of bacteria, including *C. jejuni* results in genetic selection [20]. This contrasts with the conclusion of Manning et al. [71] who concluded that some strains of *C. jejuni* are stable over long periods. However, this may be an artifact of the methods they applied to measure genetic stability, which would not be able to comprehensively detect frequency changes in mutation occurrence within the genome. We examined variations that occurred in the genome of NCTC 11168-GSv independent of a host (i.e. in agar culture after four transfers). With the possible exception of one locus (Cj1295c) encoding an unknown hypothetical protein, the genome of *C. jejuni* NCTC 11168-GSv was observed to be stable in culture after four transfers (Table 1). The genome of isolate of NCTC 11168-GSv obtained from ATCC by our group was determined to be very similar to the whole genome sequence deposited in GenBank (NCTC 11168-GS), although not identical (Figure 1, Table 1); the culture history of NCTC 11168-GS (e.g. at ATCC) is uncertain, but is likely that the genetic differences that we observed in ATCC strain versus the genome sequence in GenBank are the result of selection during propagation of the bacterium. None-the-less, our observation of unique and relatively substantive genetic changes in *C. jejuni* 11168-GSv after passage through a person, but not in culture indicates the importance of the host in selecting for a specific genotype.

### Intestinal Colonization of Mice

IL-10 KO mice were inoculated with *C. jejuni* K12E5, a single arbitrarily-selected isolate recovered from the person infected with NCTC 11168-GSv. Whole genome sequence analysis indicated that K12E5 was representative of the 52 collective isolates (Kf1 and Kf2) at the majority of the genetically variable loci examined (28 of 29). A discrepancy between Kf1/Kf2 and K12E5 was observed at only a single locus (Cj0685c); this mutation was an indel (Table 1).

**Figure 2:**
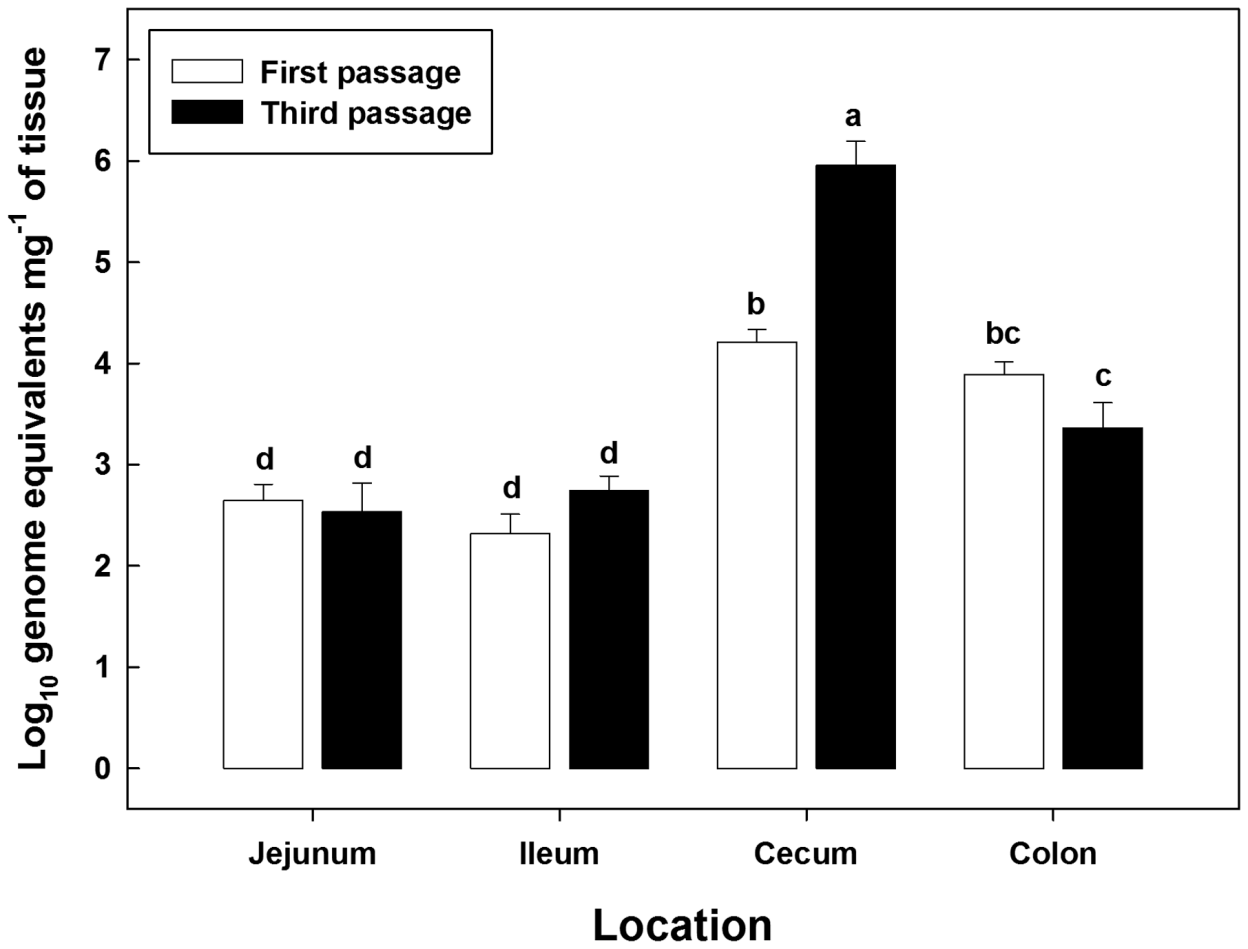
*Campylobacter jejuni* cell densities (Log_10_ genome equivalents mg^−1^ of tissue) associated with mucosa in the jejunum, ileum, cecum, and descending colon of inoculated mice. White bars indicate *C. jejuni* cell densities in the first passage, and black bars indicate cell densities in the third passage. Vertical lines associated with histogram bars represent standard errors of the mean, and bars not accompanied with the same letter differ significantly (P≤0.05).

Following inoculation of mice with *C. jejuni* K12E5, the bacterium was detected in the feces of all inoculated mice at all sample times (data not presented). Considerable quantities of *C. jejuni* associated with mucosa were detected by qPCR throughout the intestinal tracts of all inoculated mice regardless of passage (Figure 2). Densities of *C. jejuni* cells in the large intestine were higher (P≤0.021) than in the small intestine. An interaction (P<0.001) was observed between intestinal location and passage number, and this was attributed to the higher density (P<0.001) of mucosal colonization by *C. jejuni* in the cecum of mice in passage three relative to passage one. There was no difference in densities of *C. jejuni* cells between the single and triple passages at other locations. The cecum of mice is readily colonized by *C. jejuni*, and *C. jejuni* colonization density has been shown to affect both bacterial load and the composition of bacterial community without inciting inflammation [72]. By estimating *C. jejuni* NCTC 11168 cell densities in feces, a previous study suggested that colonization of the host by *C. jejuni* was enhanced with successive passages [59]. However, in contrast to our observation of enhanced colonization of the cecal mucosa, the earlier study did not observe increased *C. jejuni* cell density associated with mucosa of the jejunum, cecum, or colon. Furthermore, we observed that *C. jejuni* DNA was detected at all locations in all mice; this difference with a previous observation [59] is likely due to our use of qPCR opposed to culture-based enumeration (i.e. logistical advantages and increased sensitivity). In addition to enhanced colonization success, serial passage was also associated with a higher prevalence of enteritis as well as an increased severity of disease in IL-10 KO mice [59]. As indicated previously, we did not observe any evidence of inflammation in IL-10 KO mice in contrast to other research groups [55]–[59] even though the entire intestinal tract of IL-10 KO mice were readily colonized by *C. jejuni* NCTC 11168-GSv at high cell densities.

### Genetic Variation in a Murine Host

After three passages through IL-10 KO mice, major variations between mcK12E5/mfK12E5 and K12E5/Kf1/Kf2 occurred at nine loci (Cj0046, Cj0144c, Cj0170, Cj0548, 527380, Cj1087c, Cj1190c, Cj1305c, Cj1564) (Table 1). Variations unique to murine passage (i.e. relative to NCTC 11168-GSv and K12E5/Kf1/Kf2) occurred at four loci (Cj0046, Cj0548, 527378, Cj1087c). Cj0046 encodes a hypothetical protein of unknown function, whereas Cj1087c encodes a possible periplasmic protein. Evidence obtained to date indicates that Cj0144 is a core gene within the *C. jejuni* genome, and encodes a putative methyl-accepting chemotaxis signal transduction protein. Cj0548 encodes a putative flagellar capping protein (fliD), and the SNP that was observed in this gene was unique to mcK12E5. Reasons why this mutation was not observed in mfK12E5 are uncertain, but may indicate niche-specific microevolution in *C. jejuni* associated with mucosa within the cecum. The majority of the mutations that we observed in the genome of NCTC 11168-GSv have been observed previously in mice [23]–[25], [58]. However, changes at four loci (Cj0548, Cj0676, Cj1087c, Cj1190c) in the genome of *C. jejuni* passaged through IL-10 KO mice in the current study have not been observed previously to our knowledge. Of note, the mutation observed in Cj1087c represented a very prominent deletion (loss of 12 bases) (Table 1). In order to provide insights into the putative function of Cj1087c, we performed a Phyre2 analysis of the sequence. The most plausible association was with the lysostaphin-type endopeptidase LytM from *Staphlococcus aureus* [39]. This enzyme family is active on the pentaglycin bridge within peptidoglycan and involved in cell wall remodeling within gram-positive microorganisms. Although there is only 20.9% identity between the C-terminal sequences of LytM and Cj1087c, superimposition of their structural models provides two key insights into the putative function of Cj1087c. Firstly, there is complete conservation of a Zn^2+^ coordination pocket. This metal is a catalytic cofactor in LytM, which suggests that Cj1087c is also a metalloenzyme. Secondly, the truncation in Cj1087c is predicted to be located on the opposite side of the surface of the modeled protein, and results in the loss of a loop. This structural transition may be significant for function of the gene product by regulating interactions with other molecules. Additional research on the biological relevance of Cj1087c (e.g. virulence and/or host adaptation factor) is warranted.

Although no study to our knowledge has comparatively examined genetic changes in the genome of *C. jejuni* in humans, Wilson et al. [24] and Kim et al. [23] recently examined indel mutations in contingency loci within the genome of *C. jejuni* during passage through chickens relative to subsequent colonization success in IL-10 KO murine models of inflammation. Kim et al. [23] implicated specific indel mutations in the homopolymeric tracts of Cj0045, Cj0685, Cj1139, Cj1422, and Cj1426 in the genome of *C. jejuni* strains during chicken passage to be important in subsequent colonization and disease initiation in IL-10 KO mice. We also observed indel mutations in contingency genes of Cj0685, and Cj1139 in the *C. jejuni* NCTC 11168-GS genome in human and/or murine hosts, but not in Cj0045, Cj1422, or Cj1426.

### Conclusions

We observed that a variant strain of *C. jejuni* NCTC 11168-GSv infected and incited severe enteritis in a human being. Rapid and host-specific variations in the genome of NCTC 11168-GSv occurred in the infected person. In addition to confirming the plasticity of the genome of *C. jejuni* (e.g. in contingency genes within homopolymeric tracts) and that this human pathogenic bacterium is capable of rapid genetic selection, a number of host-specific mutations were identified in the genome of NCTC 11168-GSv within a human host, and that some of the variations observed in the human being reverted in mice. These data suggests that these variations are of biological relevance in human hosts and this warrants further investigation. Data also reinforces the importance of examining *C. jejuni* as a population of genotypes (i.e. a quasispecies) and not as a clonal strain [25]. From a practical perspective, extreme care must be taken in ensuring the genetic integrity of *C. jejuni* reference strains, especially those used as model strains.
